# *Microorganisms*—A Journal and a Unifying Concept for the Science of Microbiology

**DOI:** 10.3390/microorganisms2040140

**Published:** 2014-12-12

**Authors:** John A. Fuerst

**Affiliations:** *Editorial-in-Chief of Microorganisms*, School of Chemistry and Molecular Biosciences, The University of Queensland, St. Lucia 4072, Queensland, Australia; E-Mail: j.fuerst@uq.edu.au

The MDPI journal *Microorganisms* is still very young, having been launched in 2013, but the concept of the microorganism has been in use for at least a century as a unifying principle for the discipline of microbiology, which was cemented firmly by the intellectual work of Roger Stanier and colleagues in their *Microbial World* and other general microbiology textbooks and related articles from the 1950s to the 1970s [1,2]. Merging the idea of the microscopic and the very small with the older idea of an organism as a living entity or cell, the concept of a microorganism enabled a real appreciation of the microbial world as one that is amenable to study using similar tools and approaches even though representing distinctly different types of reproductive units and cell organizations. In the late 20th century following the work of Carl Woese and other molecular evolutionists, biologists came to appreciate the commonality among all organisms, all being comprised of cells that bear a remarkable similarity to one another and that share a common evolutionary ancestry, and consequently with major features of a largely shared genetic code and molecular biology. In this sense microbiology and biology as a whole became unified as they never had been before.

Though molecular sequencing of genomes and metagenomes has revealed immense diversity within the category microorganisms, now known to represent three distinct domains of life, Bacteria, Archaea and Eucarya, and perhaps hiding even more undiscovered types including a potential fourth domain according to recent suggestions [3,4], considering them together has scientific advantages. These include the nature of the associations and symbioses among members, the effects of scale on processes such as nutrition, the application of similar metagenomic methods to study of their diversity and ecology, the insights of a comparative approach to their genomics and potential evolutionary relationships, and analogies in some of their growth habits and strategies such as the mycelial habit of both actinobacteria and mycelial fungi [5]. When we consider a natural community, all three domains of cellular life often live together, and together with their respective viruses. Thus, methods first applied to bacteria and archaea for direct characterization of natural communities are increasingly being applied also to uncultured eukaryotes [6,7] and viruses [8] and even the attached viruses of uncultured eukaryotes [7] in such communities. When we talk about a microbial community, it is a community of *microorganisms* in the widest sense we must study, complete with bacteria, archaea, fungi, algae or protist varieties of eukaryotes, and all their accompanying viruses. In this spirit, uncultured microorganisms in different domains including the eukaryotes are increasingly studied within the same project from a particular habitat [9,10]. And ideally for a complete understanding we should aim at the culture of many cellular microorganisms, as well as retrieving genes of yet-to-be-cultured species from the environmental metagenome [11]. The recently proposed concept of “culturomics” [12] augmenting metagenomics for the bacterial gut microbiome [13] should be applied as much as is feasible to all cellular domains of microorganisms. In any case there is considerable potential overlap between microbial concepts [14]. Sizes of viruses and their genomes can overlap with those of bacterial cells, and some bacteria and bacterial genomes can be as large as unicellular eukaryotes [15]. Indeed, some of the smallest unicellular eukaryotes can be as small as many bacteria [16,17]. The giant mimivirus and similar dsDNA viruses, such as “mamavirus” and pandoravirus, though still dependent on host amoebae, may have genomes as large as some small bacteria or even (in the case of pandoraviruses) small parasitic eukaryotes [4,18,19,20]. They can have many genes for molecular biology processes such as protein translation normally possessed only by independently replicating cells; their genes may even bear phylogenetic relationship to ancient ancestors of eukaryotes [18] or to an ancestral fourth domain of life [21]. Such viruses may even have their own “virophage” parasitic satellites, stretching our definition of microorganisms even further [22]. The concept of virus may have to also widen to emphasize less their particulate virion phase and more their “virus factory” cellular phase of replication as the genuine organismal one [23]. Separation of microbiologists into distinct disciplinary camps to study microorganisms may have some advantages of specialization but also disadvantages of lack of comparative insight accompanying breadth of vision of more than one class of microorganism. Such insight can even change definitions of what comprises living organisms [24].

With the discovery of proteins such as FtsZ in bacteria of the same general family as eukaryotic tubulins of the cytoskeleton, only one example of many eukaryote homolog cytoskeletal proteins in bacteria [25], and even microtubule-forming proteins much closer to eukaryotic tubulins in some bacterial divisions [26,27], and of the ESCRT (endosomal sorting complex required for transport) III-like proteins homologous to those used in late stages of eukaryotic endocytosis used for cell division in domain Archaea in place of the FtsZ-dependent bacterial system [28], the boundaries in cell biology of distinct types of microorganism such as eukaryotes, bacteria and archaea become blurred. Similarities are such between archaeal and eukaryote cell biology and gene sequences that it is now plausible that a common ancestor of archaea and eukaryotes or even a member of an ancient archaeal lineage may have been the proto-eukaryote [29,30,31]. Additionally, a central tenet of fusion or symbiotic hypotheses for eukaryote origins is the apparent (though somewhat controversial) chimeric nature of eukaryote genomes regarding domain affinities of many genes [32,33,34,35,36], while apparent (and again controversial) widespread horizontal gene transfer (HGT) challenges the “vertical” tree concept for microbial phylogenies [29,37,38,39,40]. Synthesis of secondary metabolites such as polyketide antibiotics and anti-cancer compounds in fungi follows similar pathways to synthesis of polyketides in actinobacteria and ancient gene transfer between these distinct phyla in two domains may account for this [41]. Though domain distinctions remain workable [42,43], even in face of apparent substantial past HGT exchanges between domains, our understanding of each domain is only enhanced by what we learn of the others—understanding one microbe helps the understanding of another regardless of type.

As indicated by recent advances in understanding the phage resistance system of CRISPR-Cas [44,45,46] and the role of viruses of microorganisms in natural ecosystems and population microbiology at a global scale [47,48,49], understanding bacteria, archaea, and eukaryote microorganisms including their genomes and their ecology also needs understanding of their viruses and interactions with them at a molecular level. Such a lesson was of course latent in the initial early discovery of lysogeny in bacteria. Understanding eukaryote and other domain evolution requires an understanding of bacterial and archaeal genes and phylogeny as well as potential viral contributions to ancient molecular biology [50]. No microorganism can now be considered irrelevant to another no matter what the distance of their relationship. In addition, such insight may have quite innovative biotechnology implications as in genome engineering systems based on CRISPR/Cas [51].

Narrow specialization can open up to a wider vision, to the benefit of the individual problem areas as well as the enrichment of a broad biology of the very small, and indeed, biology of all organisms in the tree or net of life. “Microorganisms” is perhaps the umbrella for unicellular and sub-cellular life least subject to theoretical assumptions or controversies regarding phylogenetic relationships, the exact boundaries of Domains, and trees *vs.* networks, since criteria for inclusion does not depend on our view of these relationships—it therefore opens vistas to new insights even about such relationships.

The topics recently published in *Microorganisms* and now open and planned for submission soon reflect this broad vision at a high scientific level. We started with a mix of specialized *vs.* wide topics of Biology of Dinoflagellates: Advances in the Last 25 Years (1987–2012), including a review by that major pioneer of bioluminescence research and (via luciferase research) a pioneer also of a new field of bacterial quorum sensing, J. Woodland Hastings of Harvard University, and Advances and New Perspectives in Microbial Research, including a perceptive and useful critique of phage electron microscopy by Hans-W Ackermann of Laval University in Canada, and other excellent articles ranging from antibiotics from seaweed to probiotics to fungal mycotoxins in cereals to new “big data” bioinformatics analysis software to multi-resistant Gram-negative bacteria in a hospital ward to production methods for vaccinia virus in biotechnology. This issue is a very good example of the how the aims of this journal can appear in practice.

Our new topics still open for submissions include: Host-Gut Microbiota Metabolic Interactions (http://www.mdpi.com/journal/microorganisms/special_issues/gut_microbiota_metabolic_interactions), Microbial Activity in Food (http://www.mdpi.com/journal/microorganisms/special_issues/microbial_activity_food), Extremophiles (http://www.mdpi.com/journal/microorganisms/special_issues/_extremophiles), and Microbial C1 Metabolism (http://www.mdpi.com/journal/microorganisms/special_issues/microbial-C1 ) and we have more exciting and diverse topics in the planning process. We have some excellent guest editors for these special issues, including for Extremophiles, Ricardo Amils of the Severo Ochoa Centre for Molecular Biology and the NASA-affiliated Centro de Astrobiología (INTA-CSIC) in Spain, and for Microbial C1 Metabolism, Ludmila Chistoserdova and Marina G. Kalyuzhnaya of the University of Washington, Seattle, USA, internationally renowned for their studies on bacterial methane utilization and its evolution and ecology. A Special Issue on “Diversity and Dynamics of Marine Microbial Communities” (http://www.mdpi.com/journal/microorganisms/special_issues/microbial_marine) is to be edited by Professor Dr. Johannes T. Imhoff , who leads the research unit on Marine Microbial Resources at IFM-GEOMAR Helmholtz Centre for Ocean Research in Kiel, Germany.

We can see by the breadth of topics that our Special Issues cover diverse areas of contemporary fundamental and applied fields dealing with microorganisms. We encourage researchers and thinkers in the science of microorganisms to submit to these and future special issues of Microorganisms most relevant to their area of expertise. *Microorganisms* in 2015 and beyond—an exciting prospect for communicating the science of all of microbial life!
